# How well can we simulate complex hydro‐geomorphic process chains? The 2012 multi‐lake outburst flood in the Santa Cruz Valley (Cordillera Blanca, Perú)

**DOI:** 10.1002/esp.4318

**Published:** 2018-01-15

**Authors:** Martin Mergili, Adam Emmer, Anna Juřicová, Alejo Cochachin, Jan‐Thomas Fischer, Christian Huggel, Shiva P. Pudasaini

**Affiliations:** ^1^ Institute of Applied Geology University of Natural Resources and Life Sciences (BOKU) Peter‐Jordan‐Straße 82 1190 Vienna Austria; ^2^ Geomorphological Systems and Risk Research, Department of Geography and Regional Research University of Vienna Universitätsstraße 7 1010 Vienna Austria; ^3^ Department of Physical Geography and Geoecology, Faculty of Science Charles University in Prague Albertov 6 128 43 Prague 2 Czech Republic; ^4^ Department of the Human Dimensions of Global Change, Global Change Research Institute Academy of Sciences of the Czech Republic Bělidla 986/4a 603 00 Brno Czech Republic; ^5^ Department of Soil Survey Research Institute for Soil and Water Conservation Zabovreska 250, 156 27, Prague 5 – Zbraslav Czech Republic; ^6^ Unidad de Glaciología y Recursos Hidricos Autoridad Nacional del Agua Confraternidad Internacional 167, Huaráz Perú; ^7^ Department of Natural Hazards Austrian Research Centre for Forests (BFW) Rennweg 1 6020 Innsbruck Austria; ^8^ Glaciology and Geomorphodynamics Group, Division of Physical Geography, Department of Geography University of Zürich Winterthurerstrasse 190 8057 Zürich Switzerland; ^9^ Department of Geophysics University of Bonn Meckenheimer Allee 176 53115 Bonn Germany

**Keywords:** r.avaflow, GLOF, high‐mountain lakes, process chain, two‐phase mass flow model

## Abstract

Changing high‐mountain environments are characterized by destabilizing ice, rock or debris slopes connected to evolving glacial lakes. Such configurations may lead to potentially devastating sequences of mass movements (process chains or cascades). Computer simulations are supposed to assist in anticipating the possible consequences of such phenomena in order to reduce the losses. The present study explores the potential of the novel computational tool r.avaflow for simulating complex process chains. r.avaflow employs an enhanced version of the Pudasaini (2012) general two‐phase mass flow model, allowing consideration of the interactions between solid and fluid components of the flow. We back‐calculate an event that occurred in 2012 when a landslide from a moraine slope triggered a multi‐lake outburst flood in the Artizón and Santa Cruz valleys, Cordillera Blanca, Peru, involving four lakes and a substantial amount of entrained debris along the path. The documented and reconstructed flow patterns are reproduced in a largely satisfactory way in the sense of empirical adequacy. However, small variations in the uncertain parameters can fundamentally influence the behaviour of the process chain through threshold effects and positive feedbacks. Forward simulations of possible future cascading events will rely on more comprehensive case and parameter studies, but particularly on the development of appropriate strategies for decision‐making based on uncertain simulation results. © 2017 The Authors. Earth Surface Processes and Landforms published by John Wiley & Sons Ltd.

## Introduction

High‐mountain areas are often characterized by tightly coupled process domains. Gravitational mass movements starting in higher areas may impact distant lower areas not only directly, but also through cascading effects sometimes involving water bodies such as high‐mountain lakes, or through amplification by entrainment of basal material. Process chains have led to major disasters in recorded history: the 1963 Vajont landslide and flood disaster in the Italian Alps (Genevois and Tecca, 2013), the 1970 earthquake‐triggered Huascarán avalanche in the Cordillera Blanca, Peru (Evans *et al*., 2009) or the 2002 Kolka–Karmadon ice‐rock avalanche in the Russian Caucasus (Huggel *et al*., 2005) are only some examples.

Process chains often occur related to a changing cryosphere (Evans and Delaney, 2014; Haeberli and Whiteman, 2014). Climate‐induced environmental changes such as glacier retreat, the associated formation of hanging glaciers, the exposure of steep, debuttressed rock or moraine walls, and the degradation of mountain permafrost facilitates the release of initial mass movements (Alean, 1985; Haeberli, 1992; Harberli *et al*., 1997; Huggel *et al*., 2003, 2010, 2012; Noetzli *et al*., 2006; Gruber and Haeberli, 2007; Harris *et al*., 2009; Ravanel and Deline, 2011; Krautblatter *et al*., 2013; Haeberli *et al*., 2017). Also the formation and evolution of glacial lakes – most often associated with retreating or downwasting glaciers – may act as the basis for the occurrence of process chains. Plenty of literature on glacial lakes and related hazards is available for many high‐mountain areas worldwide (Hewitt, 1982; Haeberli, 1983; Richardson and Reynolds, 2000; Huggel *et al*., 2003; Breien *et al*., 2008; Hewitt and Liu, 2010; Bolch *et al*., 2011; Gruber and Mergili, 2013; Mergili *et al*., 2013; Clague and O’Connor, 2014; Emmer *et al*., 2015).

The current key research challenges include the understanding of processes at the boundary of individual mass flows as well as their physically based modelling. For instance, despite substantial progress in research on landslide generated impact waves and tsunamis the understanding of wave generation in dependence of the characteristic impacting mass movement in high mountains is still limited (Westoby *et al*., 2014). Similarly, key flood hazard parameters such as lake overtopping water volume and discharge can currently be poorly constrained. As shown in recent studies a critical challenge is the downstream propagation of increasing uncertainties of the parameter space, i.e. the uncertainties of initial landslide characteristics substantially increase downstream because of accumulating uncertainties at each process boundary (Schaub *et al*., 2016).

Well documented cases of process chain events are therefore of great importance to advance the understanding.

Several cases of mass movement‐induced glacial lake outburst floods (GLOFs) have been documented in the previous decades. This is particularly true for the Cordillera Blanca, Peru, where the communities are highly vulnerable to this type of event (Carey, 2005). Emmer *et al*. (2016a) have identified 882 high‐mountain lakes there, some of which are potentially hazardous (Vilímek *et al*., 2005; Emmer and Vilímek, 2013, 2014; Iturrizaga, 2014). A failure of the moraine dam of Lake Palcacocha in 1941 – induced by an ice avalanche or glacier calving, but possibly also by piping – caused many fatalities (Broggi, 1942; Oppenheim, 1946; Concha, 1952). More recently, GLOFs have occurred at Lake Safuna Alta in 2002 (triggered by a rock avalanche; Hubbard *et al*., 2005), at Lake Palcacocha in 2003 (landslide‐triggered dam overtopping; Vilímek *et al*., 2005), and at Lake 513 in 2010 (triggered by an ice avalanche; Carey *et al*., 2012). In 2012, a landslide from a steep moraine wall plunged into Lake Artizón Alto and triggered a process chain involving four lakes and substantial entrainment of material in the Artizón and Santa Cruz valleys (called 2012 Santa Cruz event).

Even though some of the lakes in the Cordillera Blanca have been equipped with technical and structural protection measures (Oppenheim, 1946; Zapata 1978; Portocarrero, 1984; Carey, 2005; Portocarrero, 2014; Emmer *et al*., 2016b), managing the risks associated with GLOFs in an appropriate way still remains a major challenge (Carey *et al*., 2014). The anticipation of high‐mountain process chains – and therefore also hazard mapping and zoning – largely rely on computer simulations based on mass flow models. Effectively single‐phase flow models, considering either solid or fluid, or a mixture of both, have been theoretically developed during the past decades, and more recently have also been increasingly implemented in computer codes (Voellmy, 1955; Savage and Hutter, 1989; Iverson, 1997; Takahashi *et al*., 1992; Pitman *et al*., 2003; McDougall and Hungr, 2004; Pudasaini and Hutter, 2007). Software packages such as RAMMS (Christen *et al*., 2010) or FLO‐2D have been applied to GLOF simulations (Mergili *et al*., 2011). Schneider *et al*. (2014) and Worni *et al*. (2014) have coupled different numerical models for simulating mass movement – GLOF process chains. Single‐phase models, however, cannot describe the solid and fluid phase interactions, nor the dynamic interaction between a landslide and a lake, and the consequences thereof (propagation of displacement wave) without major workarounds (Gabl *et al*., 2015). Worni *et al*. (2014) have therefore emphasized the need for more integrated concepts. Such approaches would have to rely on two‐phase flow models considering separately the solid and the fluid phase, but also the strong interactions between the phases. Pudasaini (2012) proposed such a general two‐phase flow model. This model has been applied to simulate generic examples representing submarine landslides and particle transport in lakes (Kafle *et al*., 2016), and GLOFs (Kattel *et al*., 2016). The computational tool r.avaflow (Mergili *et al*., 2017) employs an enhanced version of the Pudasaini (2012) model. Appropriate, well‐documented and well‐understood case studies are needed to test the suitability of this novel model and its computational implementation for real‐world events. In this sense, the present article aims to:

1. evaluate the suitability, potential and challenges of the novel computational tool r.avaflow with regard to the simulation of multi‐lake outburst floods, employing the 2012 Santa Cruz event as the case study;
2. based on the outcomes, identify the general key challenges process chain simulation efforts have to deal with.

Thereby we rely on a detailed analysis of the hydro‐geomorphic characteristics and processes of this multi‐lake outburst flood, outlined in the following section. Then we introduce the computational framework for two‐phase mass flows, r.avaflow. We outline the data employed for multi‐lake outburst modelling and the parameterization and modelling strategy in the 4^th^ section, before the sections presenting, and discussing our results. Finally, in the 7^th^ section we conclude with the key messages of the work.

## The 2012 Santa Cruz event

### The Santa Cruz and Artizón catchments

**Figure 1:**
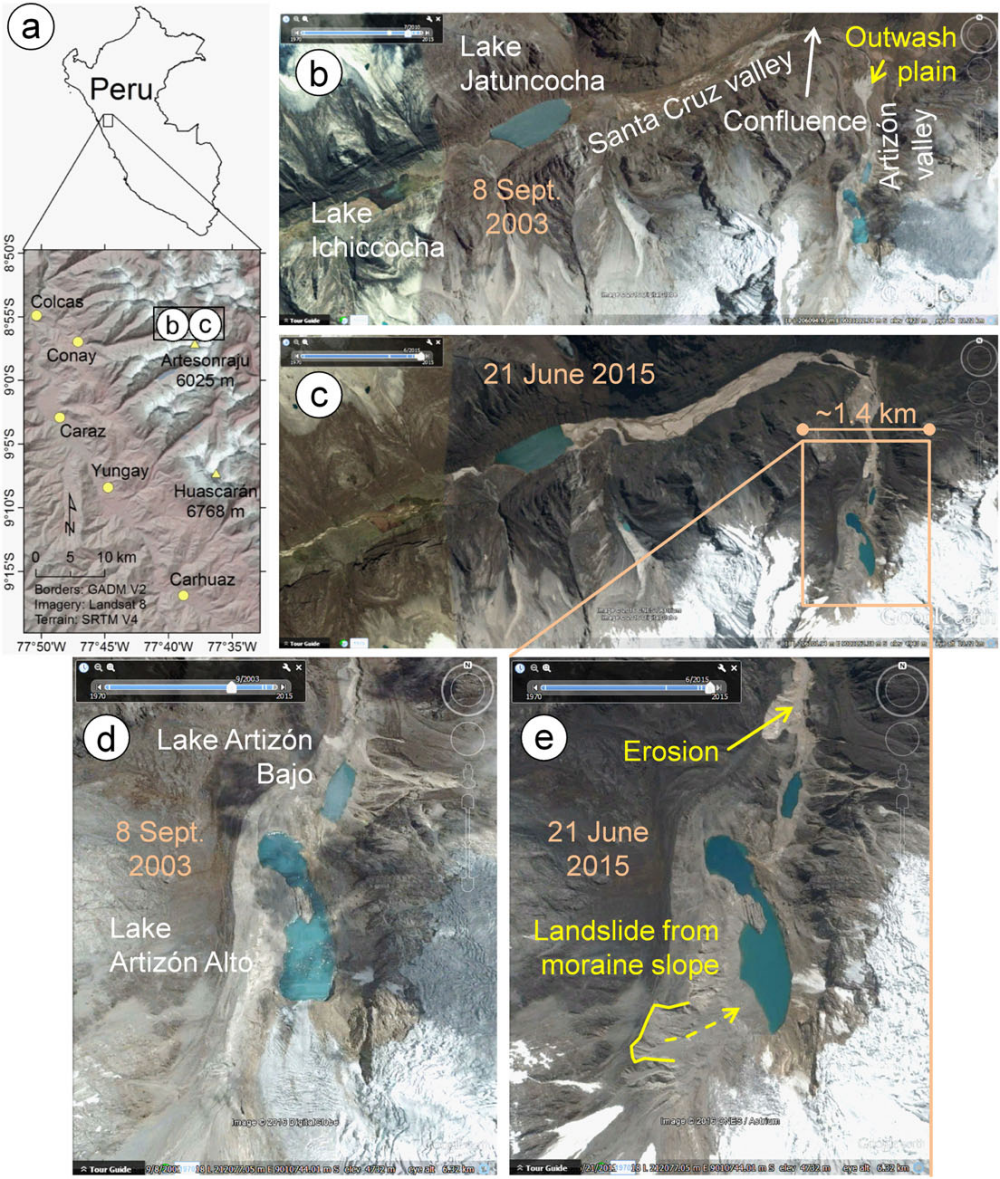
Study area before and after the event under investigation. (a) General location; (b) Santa Cruz asnd Artizón valleys before the 2012 event; (c) Santa Cruz and Artizón valleys after the 2012 event; (d) upper part of the Artizón valley before the 2012 event; (e) upper part of the Artizón valley after the 2012 event. [Colour figure can be viewed at http://wileyonlinelibrary.com]

The Santa Cruz valley is located in the Río Santa catchment in the northern part of the Cordillera Blanca, Peru (Figure 1). It occupies an area of 231 km^2^ and extends over a vertical distance of more than 4200 m between Nevado Santa Cruz (6241 m a.s.l.) and the confluence with the Santa river at Colcas (1965 m a.s.l.). Three other summits along the catchment boundary peak above 6000 m a.s.l.: Nevado Quitaraju (6036 m a.s.l.) and Pucajirca Oeste (6039 m a.s.l.) in the north and Artesonraju (6025 m a.s.l.) in the south. Most glaciers terminate at approximately 4800 m a.s.l. while large Little Ice Age (LIA) moraines are found at approximately 4400 m a.s.l., such as the moraine dams of the lakes Quitacocha, Arhueycocha and Taullicocha. The zone between 4400 m and 4800 m a.s.l. has recently been deglaciated, so that steep slopes are considered particularly susceptible to different types of mass movements (in the sense of Haeberli, 2013; Haeberli *et al*., 2017). Two large debris cone‐dammed lakes are situated in the gently‐sloped middle part of the Santa Cruz valley (lakes Ichiccocha and Jatuncocha; Table 1) and several mostly small glacial lakes have formed in hanging tributary valleys.

**Table 1:** Basic characteristics of the lakes involved in the 2012 event

Longitude Latitude	Dam type	Catchment area (km^2^)	Lake level elevation (m a.s.l.)	Surface area (10^3^ m^2^)	Volume (10^6^ m^3^)	Max. depth (m)	Reference
Artizón Alto 77.6165 W 8.9482 S	Bedrock dam	3.6	4639	138	1.42	24.6	Huaman (2001); Cochachin and Torres (2011)
Artizón Bajo 77.6152 W 8.9438 S	Moraine dam covered by debris cone accumulation	4.8	4477	32	0.36	23.3	Cochachin and Torres (2011)
Jatuncocha 77.6585 W 8.9287 S	Debris cone dam with artificial outlet	71.8	3870	501	7.22	23	Zapata (1978)
Ichiccocha 77.6781 W 8.9376 S	Debris cone dam	82.2	3828	243	Not measured (partial marshland)	Not measured (partial marshland)	Zapata (1978)

The Artizón valley (Figure 1) is a left‐side tributary to the Santa Cruz valley. The confluence is located at 3985 m a.s.l., while the highest point of the Artizón catchment is Artesonraju (6025 m a.s.l.), so that the catchment extends over a vertical distance of more than 2000 m. The Artizón catchment has an area of 8.1 km^2^. An area of 3.0 km^2^ (37% of the catchment) was occupied by glaciers in 2015, while an area of 5.6 km^2^ (69.1% of the catchment) was occupied by glaciers back in 1948. In 2016, two lakes were situated in Artizón valley: Artizón Bajo and Artizón Alto (Table 1).

### Situation before 2012

**Figure 2:**
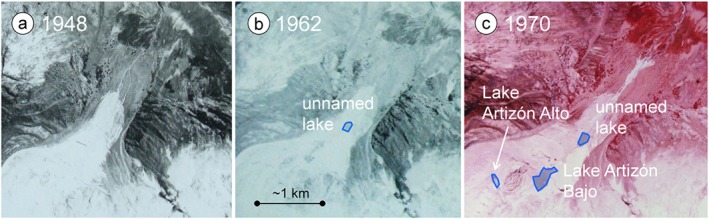
Deglaciation of the Artizón valley between 1948 and 1970 and formation of the lakes Artizón Alto and Artizón Bajo. Source: Servicio Aerofotogramétrico Nacional. [Colour figure can be viewed at http://wileyonlinelibrary.com]

Analyzing three sets of historical aerial photographs (1948, 1962 and 1970) reveals that none of the lakes in the Artizón valley existed in 1948: an unnamed lake downstream from today's Lake Artizón Bajo appeared on the 1962 and 1970 images, but disappeared later. The lakes Artizón Bajo and Artizón Alto first appeared on the 1970 image (Figure 2).

On 20 May 1997, a debris flow deposit from the right bank blocked the outlet of Lake Artizón Bajo, resulting in an increase of the lake level. This temporary blockage breached immediately, producing a GLOF‐induced debris flow which deposited in the Santa Cruz valley close to Lake Jatuncocha. Part of the Santa Cruz trekking path was damaged (Zapata, 2002). Reflecting this event, Huaman (2001) considered the lakes in the Artizón valley as hazardous and suggested the implementation of remediation measures. Cochachin and Torres (2011) described Lake Artizón Alto as hazardous and indicated that the recently deglaciated, very steep left lateral moraine alongside the lake would be unstable. Considering the conditions before 2012, Emmer and Vilímek (2014) retrospectively identified a high GLOF susceptibility for Lake Artizón Alto. The situation before 2012 is shown in Figure 1(b) and (d).

### The 2012 multi‐lake outburst flood

**Figure 3:**
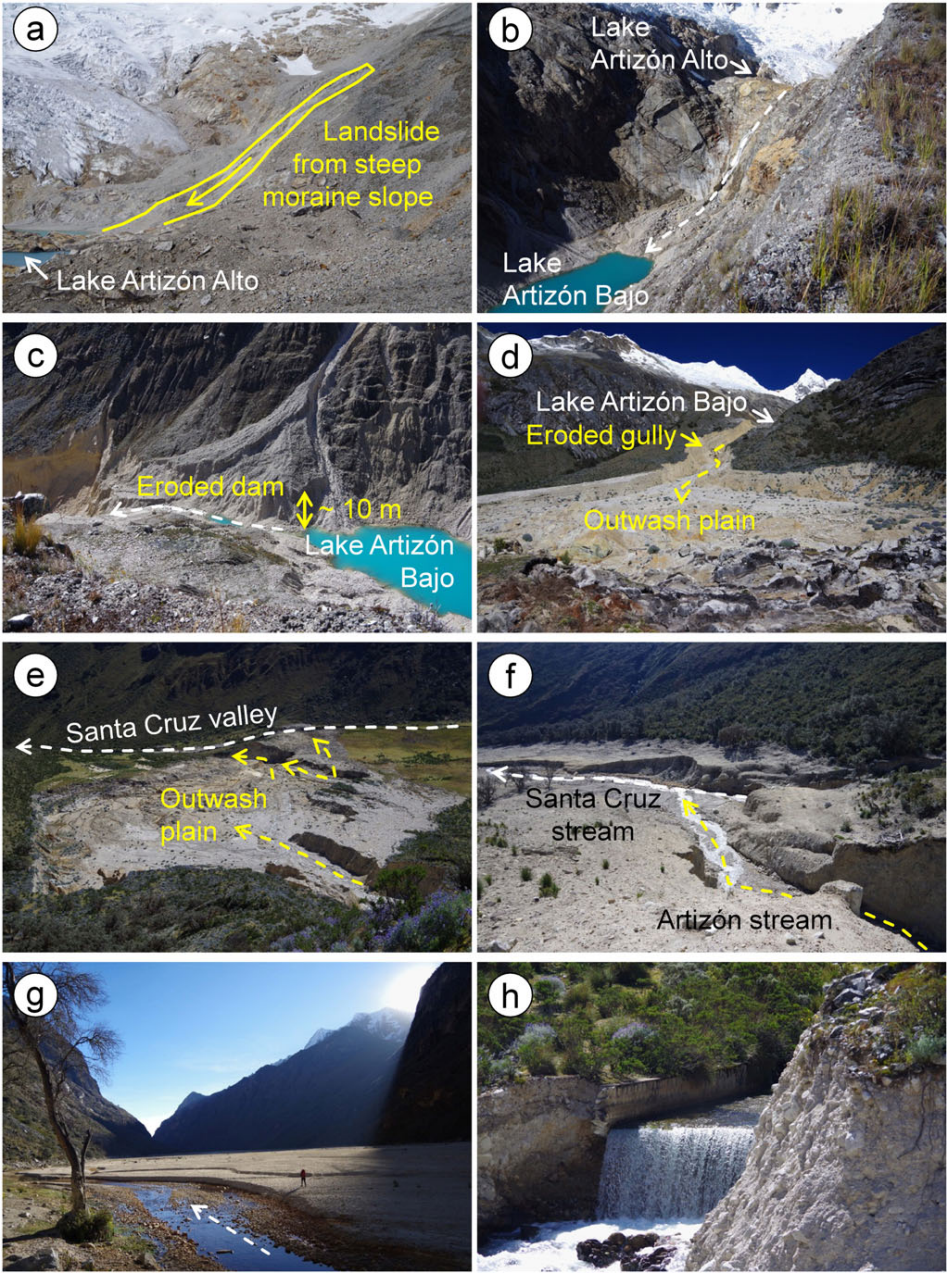
The multi‐lake outburst flood of 8 February 2012. (a) Steep moraine slopes surrounding Lake Artizón Alto with the 2012 landslide; (b) bedrock step between the lakes Artizón Alto sand Artizón Bajo, impounding lake Artizón Alto; (c) failed dam of Lake Artizón Bajo; (d) middle part of the Artizón valley leading into an outwash plain; (e) outwash plain; (f) confluence of Artizón and Santa Cruz streams; (g) Santa Cruz valley floor covered by fine‐grained deposits; (h) damaged outlet of Lake Jatuncocha. Photos: Adam Emmer, 7 and 8 July 2015. [Colour figure can be viewed at http://wileyonlinelibrary.com]

**Figure 4:**
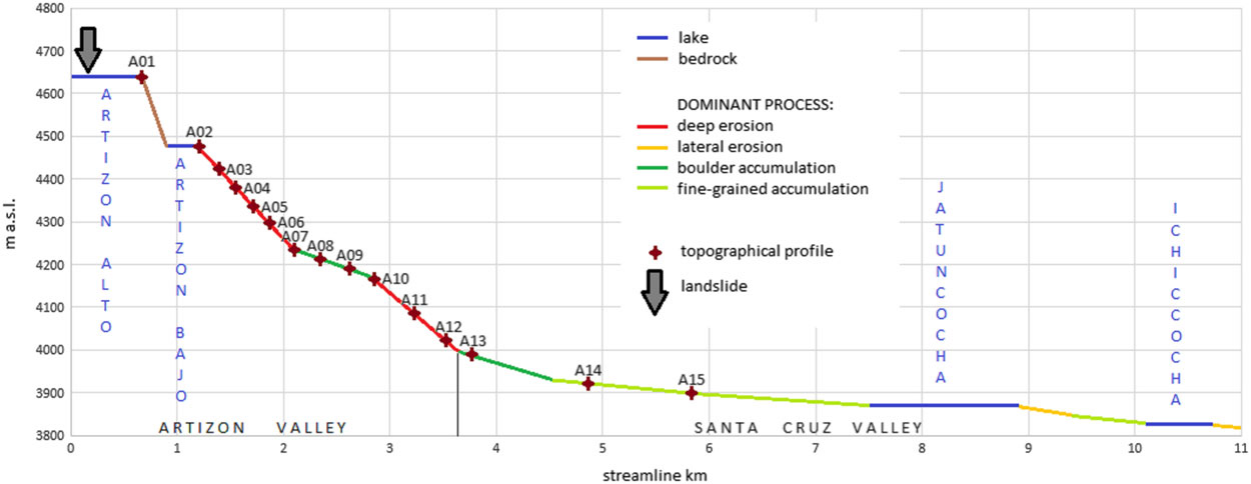
Longitudinal profile through the Artizón and Santa Cruz valleys with the dominant processes and the locations of the cross‐profiles between Lake Artizón Alto and Lake Ichiccocha. [Colour figure can be viewed at http://wileyonlinelibrary.com]

On 8 February 2012, the Artizón and Santa Cruz valleys were affected by a multi‐lake outburst flood involving two lakes in the Artizón valley and two lakes in the Santa Cruz valley (Figures 1 and 3; Table 1). The process chain was preliminarily described by Emmer *et al*. (2014) based on the analysis of remotely sensed images. A first field survey focusing on the Santa Cruz event was performed by the Autoridad Nacional del Agua directly after the event, while a detailed geomorphological field survey was performed in July 2015. Despite this time lag, the dominant geomorphological processes (erosion–accumulation interactions) were still identifiable in the field, due to the high magnitude of the event. The dominant geomorphological phenomena were mapped, described and roughly quantified, using detailed topographical measurements (laser inclinometer and rangemeter Trupulse E 200 with a resolution of 1°/0.01 m and an effective measuring range 0.5–1000 m without reflecting target; GPS /GLONASS positioning with +/‐ 5 m accuracy). 15 cross‐profiles were measured all over the affected valleys (approximately four profiles per stream line km in the upper part and one profile per stream line km in the lower part; Figure 4). These cross‐profiles reflect the dominant geomorphological processes, and also show clearly recognizable flood marks. These data were subsequently used for the correction of the digital terrain model (DTM) and for the spatial control of remotely sensed data‐based interpretation of dominant processes and delimitation of affected areas.

The trigger of the entire chain of events was a landslide released from the left lateral moraine into Lake Artizón Alto (Figure 1(e) and Figure 3(a)). The moraine slopes surrounding the lake are up to 200 m high and often very steep. Based on the analysis of remotely sensed images, the volume of the triggering landslide was estimated to 3–8×10^5^ m^3^ (Emmer *et al*., 2014). This relatively broad range is related to the uncertainty of the change in the bathymetry of Lake Artizón Alto after the event. Part of the landslide mass accumulated on the slopes above the lake (Figures 1(e) and 3 (a)) and on the lake shore. However, a significant mass of debris rushed into the shallower upper (southern) basin of Lake Artizón Alto. This part of the lake displayed a maximum depth up to 20 m before the event (Cochachin and Torres, 2011). The landslide impact produced a water wave overtopping the bedrock dam of Lake Artizón Alto, washing away the thin moraine cover into Lake Artizón Bajo downstream.

Lake Artizón Bajo is situated directly beneath the approximately 170 m high bedrock step impounding Lake Artizón Alto (Figure 3(b)). Dam overflow and sudden increase of discharge initiated a process of progressive dam erosion which eventually resulted in a release of approx. 300 000 m^3^ of water and caused the lake level to drop by 10 m (corresponding to the breach depth). However, lake Artizón Bajo did not empty completely, instead leaving behind a small remnant lake (Figure 3(c)), probably because of decreasing shear stresses after peak discharge, decreasing slope of the outflow channel, and bedrock at the bottom of the breach inhibiting further erosion (see also Worni *et al*., 2012). The bedrock bottom of the Artizón valley was exposed during the event at several particularly steep locations. A large amount of moraine and colluvium material was eroded from the middle section of the Artizón valley between Lake Artizón Bajo and the outwash plain in the lower part of the valley (mean slope of this section >15°; Figure 1(c), (e), Figure 3(d) and Figure 4).

Large boulders (up to 1 m in diameter) were first accumulated in the relatively gentle outwash plain (mean slope approx. 5°; Figure 3(e)). The flood wave overtopped the 20 m high banks of the outwash plain during the event, resulting in damage to the prevailing shrub vegetation. In the lower part of the outwash plain, the smooth Last Glacial Maximum (LGM) moraine ridge dividing the Artizón valley and the Santa Cruz valley was overtopped, and the flood wave reached the Santa Cruz stream 1 km upstream of the confluence (Figure 3(e)), washing away soil and vegetation (shrub) cover in the area affected. Part of the Artizón stream between the outwash plain and the confluence with the Santa Cruz stream is relatively steep (mean slope >10°). Erosion again dominated in this part of the valley, exposing the bedrock (Figure 4).

At the confluence with the Santa Cruz stream (Figure 3(f)), the flood wave spread across the up to 450 m wide Santa Cruz valley with a gentle mean slope of <2°. Accumulation of large boulders occurred directly at the confluence, while fine‐grained material was deposited along the entire 3.5 km long valley section between the confluence and Lake Jatuncocha (Figure 1(c), Figure 3(g), and Figure 4). Lake Jatuncocha retained the rest of the solid material transported. The outlet of Lake Jatuncocha equipped since 1969 with an open cut and an artificial dam resisted the increased water discharge. Only minor damage was recorded at the downstream face of the dam (Figure 3(h)). A marginal amount of material, compared with the amount transported upstream, was eroded downstream from Lake Jatuncocha and deposited in Lake Ichiccocha. The increased discharge also caused bank erosion and associated accumulation processes downstream in the Santa Cruz valley.

## The simulation framework r.avaflow

**Figure 5:**
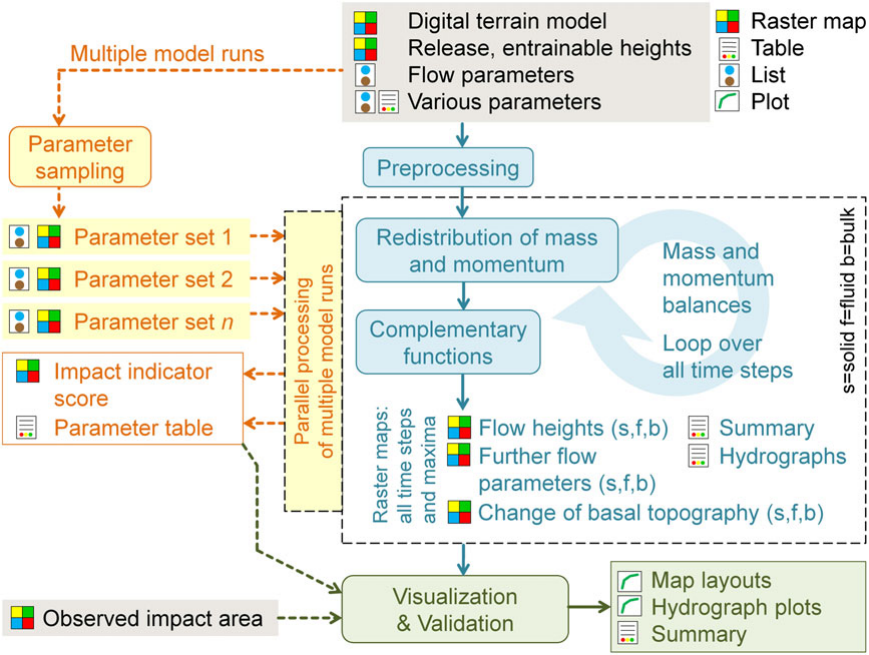
Logical framework of r.avaflow. Only those elements relevant for the present study are shown. See Mergili et al. (2017) for a comprehensive logical framework. [Colour figure can be viewed at http://wileyonlinelibrary.com]

The comprehensive GIS‐based open source computational framework r.avaflow was designed for simulating the propagation of various types of geomorphic mass flows. In contrast to most other related tools it employs a real‐two‐phase‐flow model and considers entrainment and deposition of material along the path, and is therefore suitable for the simulation of process chains and interactions. r.avaflow is described in full detail by Mergili *et al*. (2017). Essentially, only those aspects relevant for the back‐calculation of the 2012 Santa Cruz process chain are outlined in this section. The logical framework of r.avaflow is illustrated in Figure 5.

The Pudasaini (2012) two‐phase mass flow model is employed for routing mass flows from one or more defined release areas through the mountain topography given by a digital terrain model (DTM). Material can be entrained from the basal surface. Arbitrary assemblages of solid and fluid release heights and entrainable heights may be defined. r.avaflow further relies on a set of flow parameters (see Mergili *et al*., 2017 for a detailed list of input parameters). The output of r.avaflow essentially consists of raster maps – maximum and for all time steps – of solid and fluid flow heights *H* (m), velocities, pressures, kinetic energies, and entrained heights *E* (m). Optionally, solid and fluid output hydrographs are computed.

The propagation of the flow is computed through depth‐averaged mass and momentum conservation equations for the solid and fluid components. Thereby the Mohr‐Coulomb plasticity is employed for computing the solid stress, while a solid‐volume‐fraction‐gradient‐enhanced non‐Newtonian viscous stress is applied to the fluid. Generalized viscous drag and virtual mass induced by relative acceleration between the phases are employed to account for the interfacial momentum transfer. Similarly, the model includes buoyancy. Strong coupling between the solid and the fluid momentum transfers leads to simultaneous deformation, mixing, and separation of the phases. The full details of the dynamical model are given by Pudasaini (2012).

Optional complementary functions include diffusion control, conservation of volume, surface control, entrainment, and stopping (Mergili *et al*., 2017). Entrainment is computed through an empirical approach, multiplying a user‐defined, conceptual entrainment coefficient *C*_E_ (kg^‐1^) with the momentum of the flow. Solid and fluid flow heights and momenta as well as the change of the basal topography are updated in time.

r.avaflow operates on the basis of GIS raster cells. The analytical model equations are solved with the high resolution total variation diminishing non‐oscillatory central differencing (TVD‐NOC) scheme (Nessyahu and Tadmor, 1990). This scheme is commonly applied to mass flow problems (Wang *et al*., 2004). It uses a staggered grid, whereby the system is moved half of the numerical cell size with every time step.

In the present work we use the distribution r.avaflow [EXPERT] (Mergili *et al*., 2017), implemented as a raster module of the open source software package GRASS GIS 7 (GRASS Development Team, 2017), and building on the Python and C programming languages and the R software environment for statistical computing and graphics (R Core Team, 2017). Parallel processing exploiting multiple cores is enabled, facilitating multiple model runs, the computation of impact and deposition indicator scores, and parameter sensitivity analysis and optimization. r.avaflow was designed for UNIX operating systems and tested on Ubuntu 12.04, 14.04 and 16.04 LTS and Scientific Linux 6.6 (Red Hat). It is most efficiently managed on the shell script level.

The computational framework produces map layouts and animations of the main results. The model results are further validated against the observed impact area, if available. Those areas with observed mass flow impact are referred to as observed positives (*OP*), the ones without observed mass flow impact as observed negatives (*ON*). All areas with simulated mass flow impact are considered as predicted positives (*PP*), the ones without simulated mass flow impact as predicted negatives (*PN*). In the present work we relate the true positive (*TP*), true negative (*TN*), false positive (*FP*) and false negative (*FN*) predictions in order to derive the performance indicators critical success index (*CSI*), distance to perfect classification (*D*2*PC*), and factor of conservativeness (*FoC*) (Formetta *et al*., 2015; Mergili *et al*., 2017; Table 2). While *CSI* and *D*2*PC* indicate the degree of overlay between modelled and observed impact areas, *FoC* measures whether the simulation overestimates (conservative result), or underestimates the observed impact area (non‐conservative result).

**Table 2:** Performance indicators used in the present study (Formetta *et al*., 2015; Mergili *et al*., 2017)

Name	Definition	Possible range	Optimum
Factor of conservativeness (*FoC*)	$\mathrm{FoC}=\frac{\mathrm{PP}}{\mathrm{OP}}=\frac{\mathrm{TP}+\mathrm{FP}}{\mathrm{TP}+\mathrm{FN}}$	[0,∞]	1.0
Critical success index (*CSI*)	$\mathrm{CSI}=\frac{\mathrm{TP}}{\mathrm{TP}+\mathrm{FP}+\mathrm{FN}}$	[0,1]	1.0
Distance to perfect classification (*D*2*PC*)	$D2\mathrm{PC}=\sqrt{{\left(1-r_{\mathrm{TP}}\right)}^{2}+r_{\mathrm{FP}}^{2}}$	[0,1]	0.0
	$r_{\mathrm{TP}}=\frac{\mathrm{TP}}{\mathrm{OP}}$, $r_{\mathrm{FP}}=\frac{\mathrm{FP}}{\mathrm{ON}}$		

We refer to Mergili *et al*. (2017) for further details on the r.avaflow computational framework. The model codes, a user manual, a collection of test data and the associated start scripts are available at http://www.avaflow.org/.

## Simulation input

**Figure 6:**
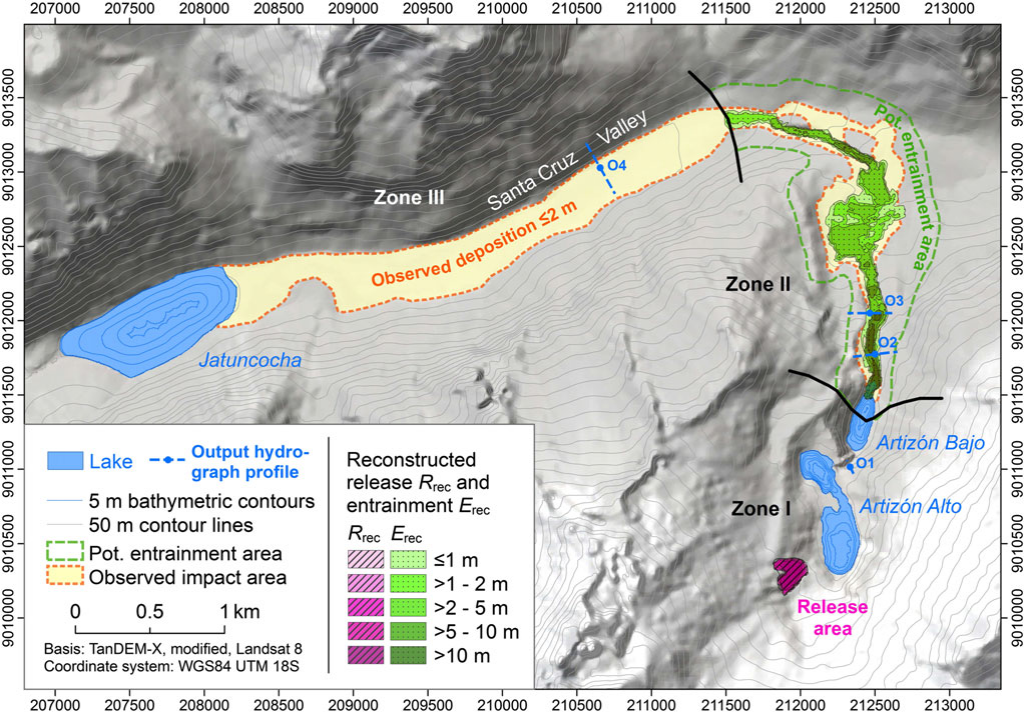
Spatial input and reference data for simulation of the 2012 Santa Cruz process chain with r.avaflow. The bold black lines separate the zones I–III. All raster maps are produced at a cell size of 5 m. [Colour figure can be viewed at http://wileyonlinelibrary.com]

We use a TanDEM‐X Digital Elevation Model (DEM) from 2013 (after the 2012 event). The DEM is available with a 10 m posting from TanDEM‐X acquisitions performed along ascending and descending orbits on 10 October 2013 and 24 January 2013, respectively, combined to reduce problems of areas masked by layover and shadow which affect differently opposite slopes along north–south oriented valleys in mountainous regions (Wegmüller *et al*., 2014). The DEM is corrected for entrainment that has occurred during the event, based on a detailed field survey, the comparison of pre‐event and post‐event Landsat 7/8 and Google Earth Pro® v. 7.1.5.1557 high resolution scenes (CNES/Astrium images), and topographic plausibility. Further, the lakes Artizón Alto, Artizón Bajo and Jatuncocha are removed by using bathymetric data (Zapata 1978; Cochachin and Torres, 2011) in order to derive a digital terrain model (DTM) displaying the basal surface of the lakes. This DTM suffers from low quality particularly in the upper section of the Artizón Valley (surrounding the lakes Artizón Alto and Artizón Bajo) so that further manual pre‐processing has to be applied (Figure 6). This step builds mainly on the well‐documented pre‐event level of the two upper lakes (Table 1), the same pre‐event Google Earth Pro® scenes as used for the estimation of entrainment, and field evidence. Given the sound basis for pre‐processing, we expect the resulting error to be manageable. However, the error cannot be quantified due to missing reference data.

The extent and depth of the release mass of the moraine slide are approximated from comparison of the same Landsat and Google Earth Pro® scenes used for the estimation of entrainment, and from field estimates of the displaced debris volume. Reconstruction yields a release volume *V*_R_ ≈ 5.3×10^5^ m^3^ and is therefore well in the range estimated (section ‘The 2012 multi‐lake outburst flood’ and Figure 6). The release height *H*_R_ is adapted accordingly for each computational experiment in order to tune the release volumes to be tested (Table 3). The lakes Artizón Alto, Artizón Bajo and Jatuncocha are considered as fluid release masses. However, they are triggered only when impacted by a moving mass. The distribution of the fluid release heights (i.e. of the lake depths) is derived from the bathymetric surveys available for all three lakes (Zapata, 1978; Cochachin and Torres, 2011). All release heights are imposed onto the DTM.

**Table 3:** Computational experiments performed with r.avaflow for the Santa Cruz process chain. In Experiment 3,*V*_R_ is varied by adapting the release height. *δ*_I_, *δ*_II_ and *δ*_III_ refer to the values of the basal friction angle of the zones I, II and III. The entrainment coefficient *C*_E_ is assumed constant throughout the potential entrainment area (Figure 6). A full list of input parameters of r.avaflow is provided by Mergili *et al*. (2017)

Experiment	Description	*V*_R_ 10^5^ m^3^	*δ*_I_ degree	*δ*_II_ degree	*δ*_III_ degree	*C*_E_ kg^‐1^	Cell size m
E1	Reference parameters	8.0	10	20	0	10^‐6.45^	10
E2*	Sensitivity to cell size	8.0	10	20	0	10^‐6.45^	5–10
E3	Sensitivity to *V*_R_ and *δ*_I_	3.0–8.0	10–18	20	0	10^‐6.45^	10
E4	Sensitivity to *δ*_II_	8.0	10	14–26	0	10^‐6.45^	10
E5	Sensitivity to *δ*_III_	8.0	10	20	0–6	10^‐6.45^	10
E6	Sensitivity to *C*_E_	8.0	10	20	0	10^‐6.55^–10^‐6.35^	10

* Experiment E2 considers only the initial part of the process chain until *t* = 100 s.

The distribution of the entrainable height of unconsolidated basal material is approximated from the reconstruction of the pre‐event topography, according to the findings of the geomorphologic field analysis and remotely sensed data. We arrive at a total entrained volume between Lake Artizón Bajo and the confluence of the Artizón and Santa Cruz valleys *E*_Rec_ ≈ 1 × 10^6^ m^3^. Also the mass deposited in the Santa Cruz Valley down to Lake Jatuncocha is estimated with *D*_Rec_ ≈ 1 × 10^6^ m^3^. Assuming that much of the initial landslide release volume was deposited within and close to Lake Artizón Alto, and some of the solid material was deposited in Lake Jatuncocha, we consider these two estimates consistent and plausible. All raster maps serving as input for r.avaflow are prepared at a cell size of 5 m and are illustrated in Figure 6. Even though the original TanDEM‐X data only support a spatial resolution of 10 m, resampling to 5 m is considered useful for exploring the influence of the computational cell size on the results (section ‘Experiment E2: initial stage of process chain, sensitivity to cell size’).

We perform six computational experiments (E1–E6) for the 2012 Santa Cruz event (Table 3). Preliminary simulations have suggested that the results in terms of the travel distance, impact area, and entrained volumes are sensitive to the uncertain initial landslide volume *V*_R_, and to the uncertain basal friction angle *δ* and entrainment coefficient *C*_E_. While these parameters and the cell size employed for the simulations are varied within and between the experiments, all other parameter values are kept constant in accordance with Mergili *et al*. (2017). The internal friction angle is *φ* = 35°. Further, all experiments build on the same set of basic assumptions:

- Both the landslide release mass and the entrainable material are in a state near or at saturation. Precipitation had been recorded at the stations nearby the days before, but the real state of saturation remains unknown and was most likely more complex. We apply rates of 70% solid and 30% fluid per volume – an estimate considered reasonable for the materials involved – throughout all the experiments E1–E6. The lakes are considered 100% fluid.
- We divide the flow path into three zones I, II and III, differing in terms of *δ* and the possibility of entrainment. Zone I covers the initial part of the process chain down to Lake Artizón Bajo, zone II the potential entrainment area down to the confluence of the Artizón and Santa Cruz Valleys, and zone III the Santa Cruz Valley downstream from the confluence (Figure 6).
- *C*_E_ is constant throughout the potential entrainment area.

We only consider the part of the process chain starting from the release of the initial landslide down to Lake Jatuncocha, disregarding the minor flood wave downstream from Lake Jatuncocha. Simulation times of *t* = 600 s (experiment E1), *t* = 100 s (Experiment E2), and *t* = 400 s (all other experiments) are applied. Experiment E1 builds on an ad hoc assumption of the key parameters (*V*_R_, *δ*, *C*_E_, cell size) in terms of qualitative and quantitative correspondence with the event documentation and computational efficiency. In the experiments E2–E6 we vary each of the assumed parameters in order to explore the sensitivity of the model results and performance and to justify the choice of the values used in experiment E1 (Table 3). The observed impact area between Lake Artizón Bajo and Lake Jatuncocha (zones II and III) is used as reference for model validation (Figure 6). The impact area in zone I is not well defined through the event documentation and therefore neglected as well as the lakes themselves and the area downstream from Lake Jatuncocha. Further, we do not evaluate the simulation results (or adapt the parameters respectively) in terms of flow velocities or travel times as no field observations of these parameters are available.

## r.avaflow simulation results

### Experiment E1: reference simulation configuration

**Figure 7:**
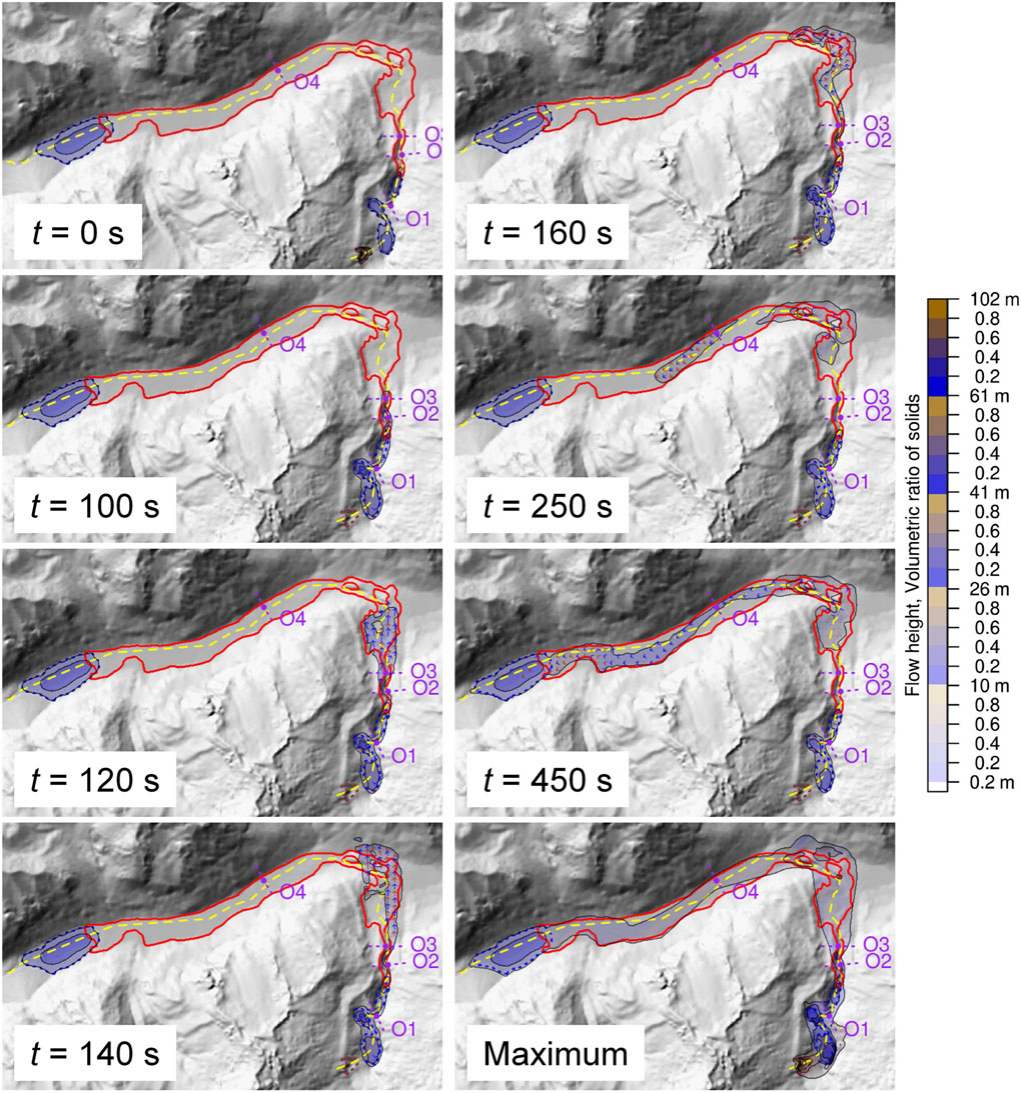
Flow height maps derived in the computational experiment E1 for selected points in time, and for the maximum over the entire simulation (*H*_Max_). The dashed yellow line represents the main flow path (defined manually from the DTM and satellite imagery), the solid red line the observed impact area of the process chain down to Lake Jatuncocha. O1–O4 represent the output hydrographs (Figure 6 and Figure 8). [Colour figure can be viewed at http://wileyonlinelibrary.com]

The results in terms of the simulated impact areas (maximum flow height *H*_Max_ ≥ 0.2 m) are shown in Figure 7 and correspond satisfactorily well to the event documentation with parameter assumptions of *δ*_I_ = 10°, *δ*_II_ = 20°, and *δ*_III_ = 0°, *C*_E_ = 10^‐6.45^ kg^‐1^, and a cell size of 10 m (Table 3 and Table 4). However, we particularly note that: (i) lateral spreading of the flow is overestimated in the Artizón valley down to the outwash plain by locally up to approximately 180 m on one side; (ii) run‐up on the slopes of the Santa Cruz valley is overestimated (locally up to 120 m, compared with very limited observed run‐up); and (iii) the simulated flow, in contrast to the observed flow, does not impact the entire width of the Santa Cruz valley all the way down to Lake Jatuncocha. Figure 7 illustrates the flow height evolution from *t* = 0 s to *t* = 450 s, and the maximum flow height throughout the simulation period. The period from *t* = 0 s until *t* = 100 s is considered in detail in experiment E2.

**Table 4:** Validation summary for the computational experiments E3–E6. Refer to Table 2 for the description of the performance indicators *CSI*, *D*2*PC*, and *FoC*. Bold letters refer to the optimized parameter set and scores (experiment E1)

Experiment E3 – *V*_R_ = 3 · 10^5^ m^3^					
*δ*_I_ (°)	*C*_E_ (kg^‐1^)	*CSI*	*D*2*PC*	*FoC*	*V*_E_ (10^6^ m^3^)
10	10^‐6.45^	0.47	0.47	0.66	0.15
15	10^‐6.45^	0.02	0.98	0.02	0.01
20	10^‐6.45^	0.02	0.98	0.02	0.00
Experiment E3 – *V*_R_ = 8 · 10^5^ m^3^					
*δ*_I_	*C*_E_	*CSI*	*D*2*PC*	*FoC*	*V*_E_
**10**	**10** ^**‐6.45**^	**0.59**	**0.20**	**1.21**	**1.09**
15	10^‐6.45^	0.34	0.60	0.60	0.32
20	10^‐6.45^	0.04	0.96	0.05	0.02
Experiment E4					
*δ*_II_	*C*_E_	*CSI*	*D*2*PC*	*FoC*	*V*_E_
14	10^‐6.45^	0.56	0.15	1.56	1.44
**20**	**10** ^**‐6.45**^	**0.59**	**0.20**	**1.21**	**1.09**
26	10^‐6.45^	0.40	0.48	0.85	0.70
Experiment E5					
*δ*_III_	*C*_E_	*CSI*	*D*2*PC*	*FoC*	*V*_E_
**0**	**10** ^**‐6.45**^	**0.59**	**0.20**	**1.21**	**1.09**
2	10^‐6.45^	0.52	0.30	1.07	1.09
6	10^‐6.45^	0.40	0.47	0.88	1.09
Experiment E6					
*δ*	*C*_E_	*CSI*	*D*2*PC*	*FoC*	*V*_E_
Raster map*	10^‐6.55^	0.58	0.19	1.25	0.76
**Raster map** *	**10** ^**‐6.45**^	**0.59**	**0.20**	**1.21**	**1.09**
Raster map*	10^‐6.35^	0.58	0.22	1.15	1.60

* *δ* is varied spatially, using the optimum values for the zones I, II, and III.

The differentiation of *δ* most probably aggregates various aspects of the complex interaction of the flow with the basal surface. On the one hand, *δ*_II_ > *δ*_I_ > *δ*_III_ results from entrainment: r.avaflow considers full momentum uptake through entrainment (Mergili *et al*., 2017), a generalization that possibly leads to an overestimate of the momentum. This effect is compensated by increasing the basal friction. On the other hand, a value of *δ*_III_ very close to zero (approximated by *δ*_III_ = 0°) is needed to propagate the flow down to Lake Jatuncocha. Such a low value can partly be justified by the interaction of the flow with a saturated valley bottom. However, in the context of this computational experiment *δ*_I_, *δ*_II_, and *δ*_III_ cannot be considered as purely physical parameters, but rather as the result of an optimization procedure, aggregating various effects (experiments E3–E5; Table 2; Discussion).

**Figure 8:**
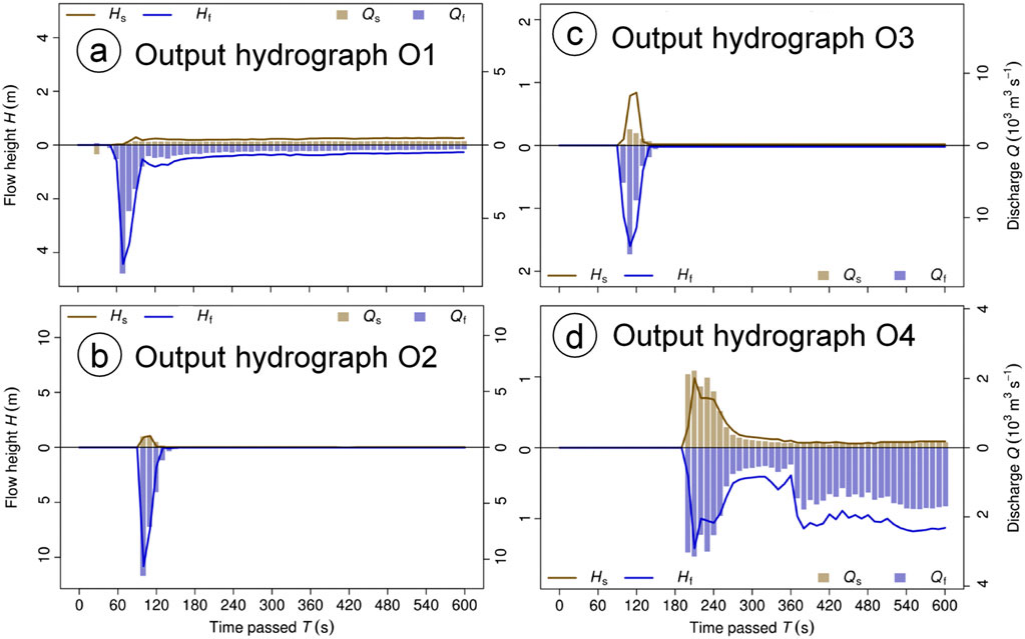
Output hydrographs O1–O4 generated in experiment E1 (see Figure 6 for the location of the hydrograph profiles). Columns represent the discharge, lines the flow height. The solid component is shown above, the fluid component below the horizontal line. Note that the fluid components are drawn in negative direction in order to enhance the readability of the figure. (a) Output hydrograph O1; (b) output hydrograph O2; (c) output hydrograph O3; (d) output hydrograph O4. [Colour figure can be viewed at http://wileyonlinelibrary.com]

The hydrographs O1–O3 (Figure 6 and Figure 8) clearly show a sudden peak in fluid and solid discharge at the point of time the flood wave passes through. The decrease of the discharge being slower than the increase reveals a strong frontal surge of solid and fluid. All three hydrographs suggest a rapid run‐through of the main flood wave (few tens of seconds), with substantial residual discharge afterwards only observed at O1. O4 displays a second, slower, increase and still records a notable simulated discharge at *t* = 600 s.

Also the effects of entrainment are documented in the output hydrographs: while the solid content in O1 originates from the initial landslide, the increasing solid fraction from O2–O4 reflects the entrainment along the flow path. With *C*_E_ = 10^‐6.45^ kg^‐1^ the simulated entrained volume is *V*_E_ = 1.09×10^6^ m^3^, corresponding well to the reconstructed entrained volume of 1×10^6^ m^3^. Therefore we consider *C*_E_ = 10^‐6.45^ kg^‐1^ a reasonable assumption. However, lowering of the dam level of Lake Artizón Bajo – and therefore also the degree of emptying of this lake – is underestimated, compared with field observations: the simulated total fluid discharge at the hydrograph O2 directly downstream from Lake Artizón Bajo sums up to approx. 250 000 m^3^, the same magnitude as the estimated 300 000 m^3^ release from the lake. However, the simulated volume is supposed to include also water from Lake Artizón Alto, and the rapid drop of the discharge after its peak to almost zero indicates a very limited degree of progressive entrainment and lowering of the dam (Figure 8(b)).

### Experiment E2: initial stage of process chain, sensitivity to cell size

**Figure 9:**
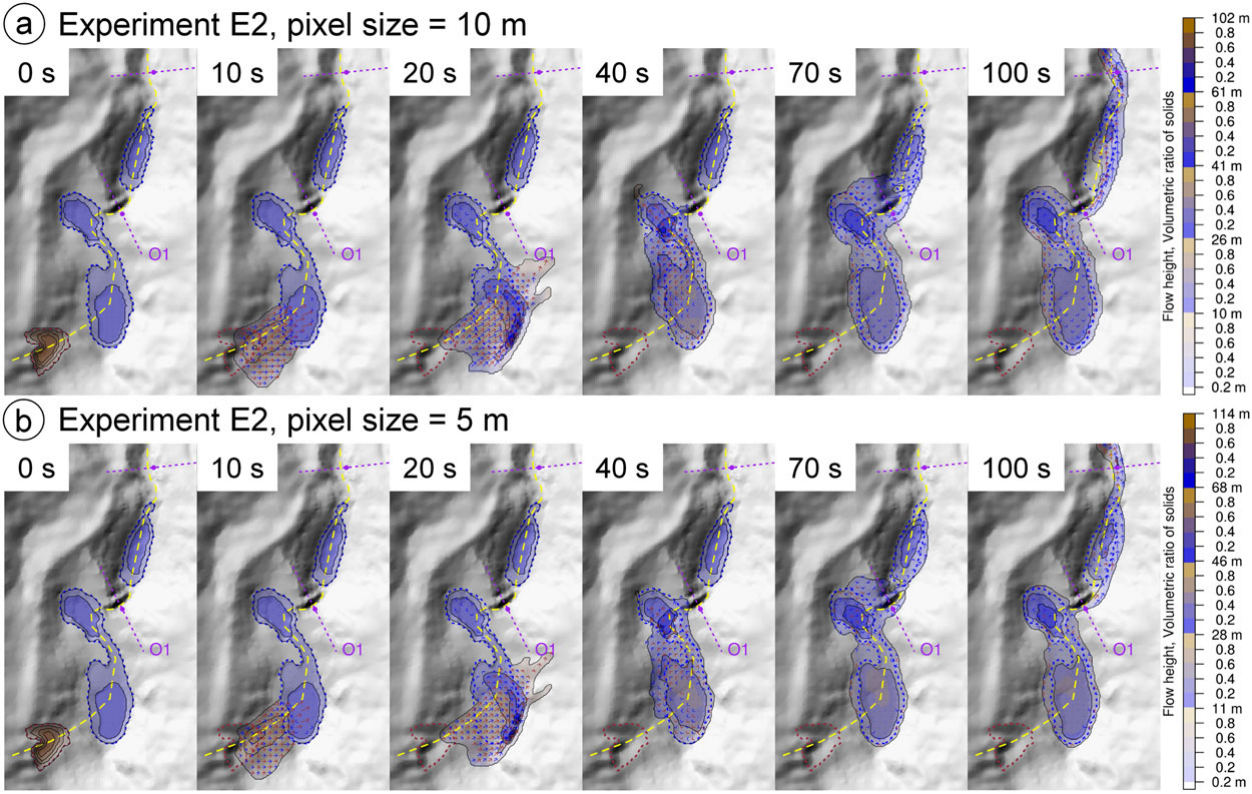
Flow height maps resulting from experiment E2 for selected points in time from *t* = 0 s to *t* = 100 s, representing the initial stage of the process chain. The dashed line represents the main flow path. (a) Simulation with 10 m cell size; (b) simulation with 5 m cell size. [Colour figure can be viewed at http://wileyonlinelibrary.com]

The flow should be a sufficient number of cells wide in order to ensure a proper numerical treatment. Even if we assume that 10 m DTM resolution is sufficient for capturing the topographic characteristics relevant for the flow, and despite a cell size of 5 m represents an oversampling, simulations at the finer resolution assist in evaluating whether 10 m resolution are also sufficient from a numeric point of view. Experiment 2 considers in detail the initial stage of the process chain (until downstream from Lake Artizón Bajo) and thereby evaluates the effect of the cell size applied to the simulation on the patterns and evolution of the flow height (Figure 9; Table 3).

The landslide reaches Lake Artizón Alto after a few seconds and causes an impact wave running up the opposite slope towards NE (*t* = 20 s). Thereby, the maximum simulated value of *H* = 102 m (10 m cell size) and 114 m (5 m cell size), respectively. The wave moves back into Lake Artizón Alto and overtops the bedrock dam of the lake. The resulting flood wave impacts Lake Artizón Bajo situated downstream (*t* = 70 s). The resulting wave in Lake Artizón Bajo overtops its moraine/debris cone dam, triggering erosion of the dam material and a debris flood propagating farther downstream (*t* = 100 s; Figure 7 and Figure 8). Except for the difference in the maximum value of *H* as well as some details in the flow patterns the simulations at 10 m and 5 m cell size yield fairly identical results. Consequently, we consider a cell size of 10 m acceptable for the purpose of the present study.

### Experiment E3: importance of the initial stage

**Figure 10:**
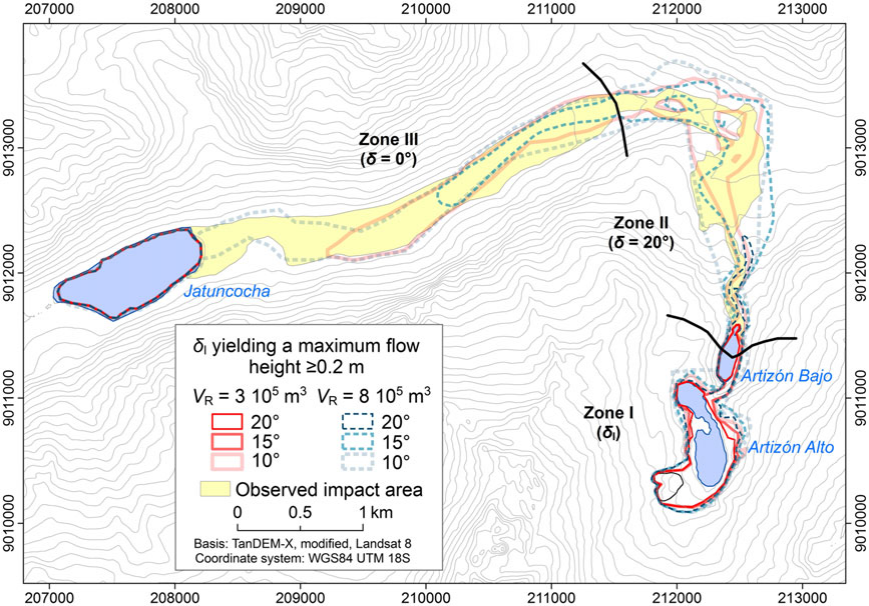
Simulated impact area (HMax ≥ 0.2 m) derived with six combinations of VR and δI (experiment E3). The observed impact area is shown as reference. [Colour figure can be viewed at http://wileyonlinelibrary.com]

**Figure 11:**
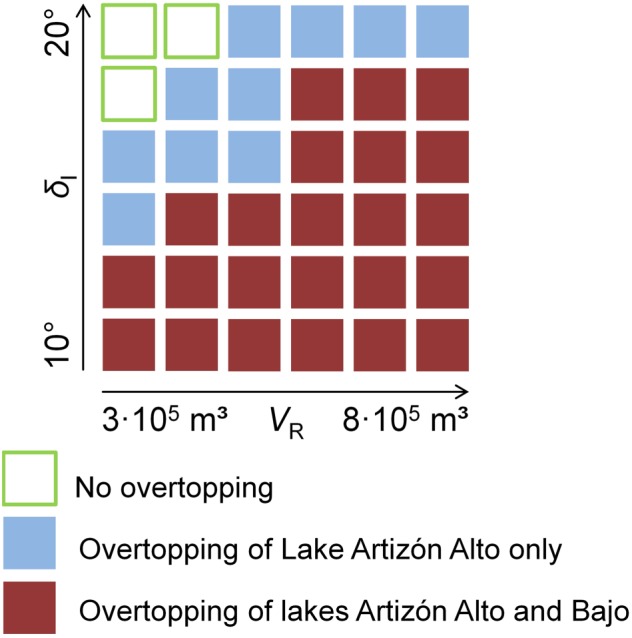
Dependency of overtopping of the lakes Artizón Alto and Artizón Bajo on the combination of the release volume of the initial landslide *V*_R_ and the basal friction angle *δ*_I_ applied to r.avaflow. [Colour figure can be viewed at http://wileyonlinelibrary.com]

Experiment E3 (Table 2) demonstrates the sensitivity of the process chain simulation to the assumptions concerning the initial stage of the phenomenon, i.e. the release volume and *δ*_I_. Figure 10 shows the simulated impact areas in terms of *H*_Max_ ≥ 0.2 m, obtained with six combinations of *V*_R_ and *δ*_I_. With the combination of *V*_R_ = 3.0×10^5^ m^3^ and *δ*_I_ = 20°, r.avaflow does not simulate overtopping of the dam of Lake Artizón Alto and, consequently, no flood wave downstream. With*V*_R_ = 3.0×10^5^ m^3^ and *δ*_I_ = 15°, and with*V*_R_ = 8.0×10^5^ m^3^ and *δ*_I_ = 20°, the flow alleviates in Lake Artizón Bajo. The simulated flood wave proceeds farther downstream with the other combinations listed in Table 4. Figure 11 illustrates the dependency of the occurrence of overtopping and the development of a process chain on the combination of *V*_R_ and *δ*_I_. The process chain reaches Lake Jatuncocha only with *V*_R_ = 8.0×10^5^ m^3^ and *δ*_I_ = 10°. Run‐up against the slopes of the Santa Cruz valley is more pronounced with larger release volumes. With *V*_R_ = 8.0×10^5^ m^3^ and *δ*_I_ ≤ 15° excessive spreading of the flow is simulated in the Artizón valley.

*V*_E_ is severely underestimated compared with the reconstruction for all experiments except *V*_R_ = 8.0×10^5^ m^3^ and *δ*_I_ = 10° (Table 4). Both *CSI* and *D*2*PC* indicate the best model performance with *V*_R_ = 8.0×10^5^ m^3^ and *δ*_I_ = 10°. This combination expectedly also yields the most conservative result (highest value of *FoC*).

### Experiment E4: sensitivity to the basal friction in zone II

**Figure 12:**
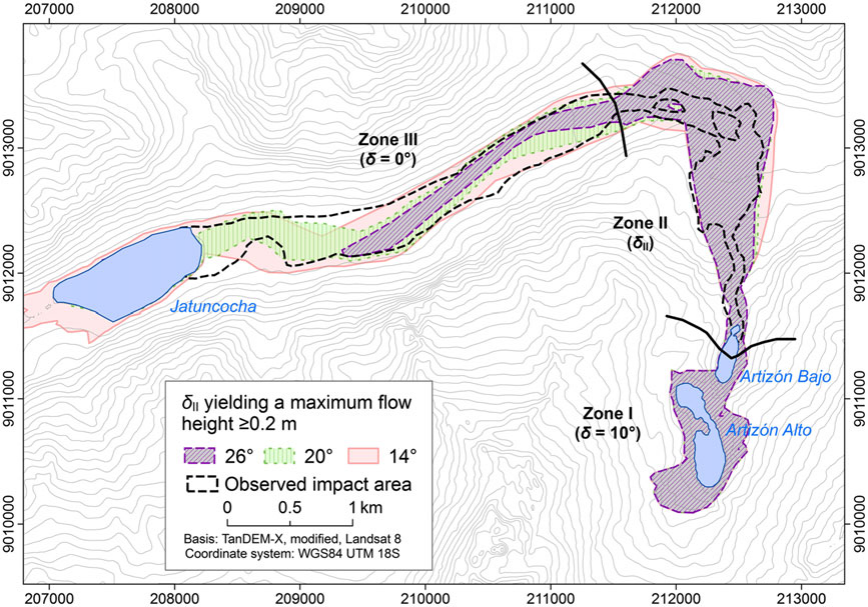
Simulated impact area (HMax ≥ 0.2 m) derived with three assumptions of δII (experiment E4). The observed impact area is shown as reference. [Colour figure can be viewed at http://wileyonlinelibrary.com]

Figure 12 illustrates the sensitivity of the distribution of *H*_Max_ on *δ*_II_ (assumption of basal friction angle in zone II), leaving *δ*_I_ and *δ*_III_ unchanged (Table 4). The flow reaches Lake Jatuncocha with *δ*_II_ ≤ 20°. Furthermore, lower values of *δ*_II_ lead to a higher degree of lateral spreading than higher values of *δ*_II_, which is reasonable. With values of *δ*_II_ = 14° r.avaflow simulates an overflow of Lake Jatuncocha before *t* = 400 s. These phenomena are reflected in *FoC* (Table 4) and are a consequence of the fact that more momentum is gained in zone II with lower values of *δ*_II_. Lower values of *δ*_II_ further induce intense entrainment in the lower part of zone II (*V*_E_ = 1.44×10^6^ m^3^). *CSI* indicates the optimum model performance at 20°, *D*2*PC* at 14° (Table 4).

### Experiment E5: sensitivity to the basal friction in zone III

**Figure 13:**
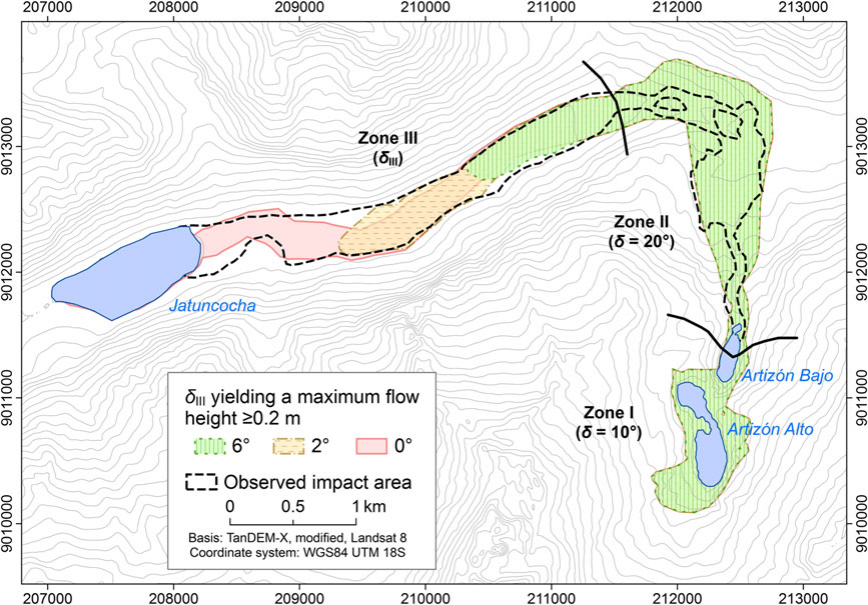
Simulated impact area (*H*_Max_ ≥ 0.2 m) derived with three assumptions of *δ*_III_ (experiment E5). The observed impact area is shown as reference. [Colour figure can be viewed at http://wileyonlinelibrary.com]

Some of the patterns observed in experiment E4 are even more pronounced in experiment E5 (Figure 13). The variation of *δ*_III_ (assumption of basal friction angle in zone III) obviously does not influence the patterns of *H*_Max_ in zone II, but strongly impacts *H*_Max_ farther downstream in zone III. The flow only reaches Lake Jatuncocha with the assumption of *δ*_III_ = 0°. Even with *δ*_III_ = 2° it stops well before Lake Jatuncocha, and with *δ*_III_ = 6° the flow proceeds less than half‐way from the confluence towards Lake Jatuncocha. The relatively flat terrain only permits a longer runout in the case of very low values of *δ*_III_. This phenomenon is also reflected in the validation scores (Table 4): *CSI* and *D*2*PC* indicate a decrease of the model performance with increasing values of *δ*_III_, *FoC* indicates a decreasing degree of conservativeness. As prescribed by the definition of the potential entrainment zone, *V*_E_ is not influenced by variations in *δ*_III_.

### Experiment E6: sensitivity to the entrainment coefficient

Within the tested range of assumptions, entrainment influences the general pattern of *H*_Max_ ≥ 0.2 m to a relatively minor degree only, so that the validation scores – except for *FoC* – are poorly differentiated (Table 4). However, with *C*_E_ = 10^‐6.35^ kg^‐1^ the flow stops approximately 150 m before reaching Lake Jatuncocha. Higher values of *C*_E_ further result in less pronounced run‐up and less lateral spreading in the Santa Cruz Valley. This pattern is most likely explained by the higher solid content induced by higher entrainment rates. Flows rich in solid are less diffusive in terms of spreading than those rich in fluid.

**Figure 14:**
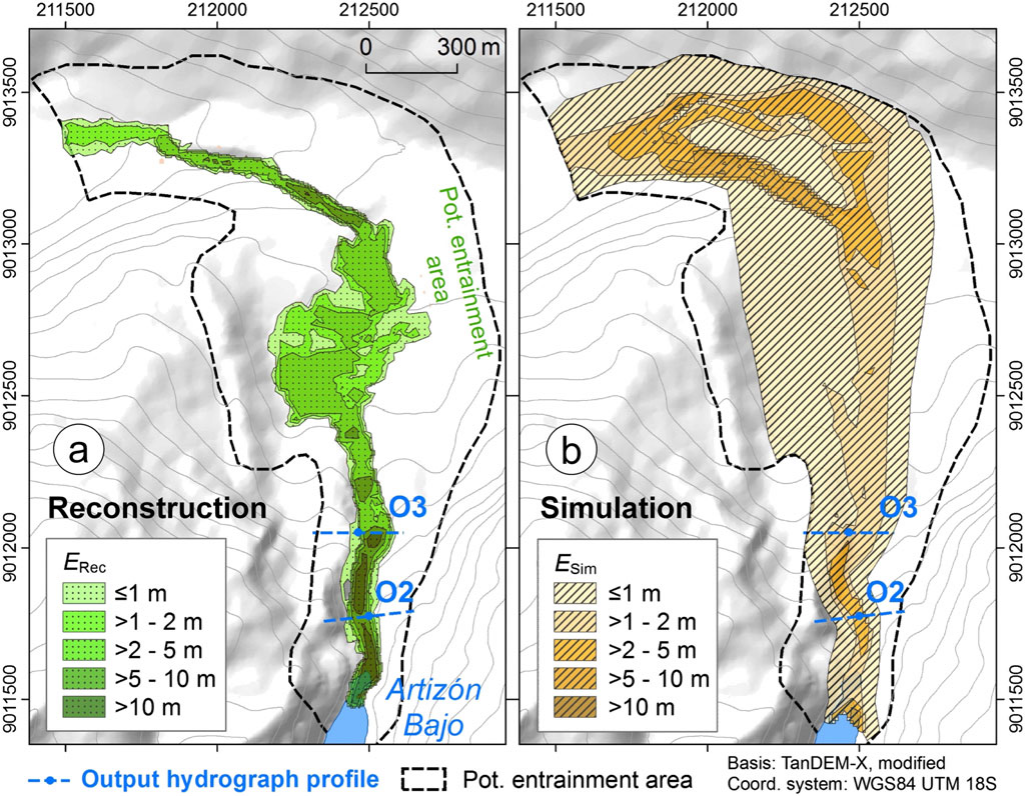
Reconstructed and simulated entrained heights in the potential entrainment area (Figure 6). (a) Reconstructed entrained height (*E*_Rec_); (b) simulated entrained height (*E*_Sim_). [Colour figure can be viewed at http://wileyonlinelibrary.com]

The zone of simulated entrainment laterally extends beyond the zone of reconstructed entrainment, reflecting the overestimated degree of spreading of the simulated flow (Figure 14; experiment E1). More importantly, entrainment in the lowermost section of the Artizón valley between the outwash plain and the Santa Cruz valley is generally overestimated, compared with the dam of Lake Artizón Bajo and the valley section directly downstream (Figure 1 and Figure 6). This phenomenon is possibly explained by a spatial pattern in the mechanical properties of the entrainable material (higher level of compaction of the older sediments deposited downstream). However, the event documentation does not support a spatial differentiation of *C*_E_.

## Discussion

Multi‐hazard contexts have been increasingly considered by the scientific communities in the past years, both on a conceptual level (Kappes *et al*., 2012), and – particularly for process chains – on the level of event back‐calculation by means of computer simulations (Schneider *et al*., 2014). Recent studies have analyzed and quantified the error range involved when simulating process chains due to limited physical understanding, limited capabilities of current models at process boundaries, and associated uncertainties concerning the surrounding surface topography, material and mass flow parameters and characteristics (Westoby *et al*., 2014; Schaub *et al*., 2016). A particular challenge concerns the coupling of different simulation models, or the integration of different cascading mass flow processes in one integrated model. Here we follow the call of Worni *et al*. (2014) for more integrated approaches. Thereby, the 2012 Santa Cruz event serves to demonstrate and highlight some of the major challenges process chain simulations are facing, particularly with regard to the data they rely on:

1. Process chains often occur in remote areas characterized by steep topography. Even though modern remote sensing techniques help to capture the situation before and after events, the quality of such data is often limited. Further, highly demanding field work is necessary to: (i) improve the understanding of the individual and interacting processes; and (ii) build a sound basis for back‐calculations.
2. As a consequence of (1), the detailed process understanding is limited and simulation input and reference data involve uncertainties. In the present study, the reconstruction of the topography was based on state‐of‐the‐art radar remote sensing technology, bathymetric data and further field observations. This is more than is typically available in remote high‐mountain areas but still implies uncertainties with respect to release and entrained depths (Figure 6) and the associated volumes. Basal friction values and entrainment coefficients are the products of back‐calculations, without a direct mechanical basis. The implementation of a physically‐based entrainment model (Pudasaini and Fischer, 2016) with r.avaflow is in progress.
3. No reference data are available to evaluate the simulated flow velocities or travel times. Therefore, parameters are optimized on the basis of observed spatial, but not temporal patterns.

Simulation results are particularly sensitive to variations of uncertain key input: initial conditions such as volume and solid content of the release and flow parameters such as the basal friction angle, or the entrainment coefficient. We note that varying the conditions in Zone I (down to Lake Artizón Bajo) may lead to a highly nonlinear behaviour (Figure 11). The reason is the fact that process chains involve threshold phenomena and strong positive feedbacks: even moderate overtopping of a dam may induce entrainment and a powerful flood wave or debris flow downstream, while there is no process chain at all in the case of no overtopping. An initial landslide of *V*_R_ = 3×10^5^ m^3^ requires a lower basal friction angle (*δ* <12–14°) to develop sufficient kinetic energy to result in overtopping of Lake Artizón Bajo than an initial landslide of *V*_R_ = 8×10^5^ m (*δ* <18–20°). While the general patterns illustrated in Figure 11 are most probably generally valid, the threshold values would shift when changing the initial conditions or numerical treatments. For input combinations with predicted overtopping of Lake Artizón Bajo, the effects downstream depend on the varied parameters in a near‐linear way (Figure 10).

Varying the initial conditions or flow parameters in the Zones II or III (i.e. after the interaction of the mass movement with the two upper lakes) generally leads to a rather linear (predictable) behaviour (Figures 12 and 13). The *FoC* (which is proportional to the simulated affected area) is most suitable to quantify this observation: varying the basal friction angle in the zones II or III leads to more or less linear changes of the *FoC*, and also of *V*_E_. Increasing *δ*_II_ from 14° to 20° and 26° leads to decreases in *FoC* by 0.35 and 0.36, respectively. This would mean that the size of the simulated impact area would decrease by 35% and 36% of the observed impact area. Increasing *δ*_III_ from 0° to 2° and 4° leads to decreases in *FoC* of 0.14 and 0.19, respectively. These patterns demonstrate that the energy gained by a flow, and therefore also its travel distance and lateral spreading, show a near‐linear relationship with the basal friction as long as no interactions with a reservoir occur. Likewise, increasing *C*_E_ in Zone II from 10^‐6.55^ to 10^‐6.45^ and further to 10^‐6.35^ leads to a relatively linear increase of *V*_E_ of 0.33×10^6^ and 0.51×10^6^, respectively. However, these near‐linear patterns have to be interpreted with caution as the processes involved may be nonlinear: the empirical entrainment model applied with r.avaflow represents a rough approximation only, which will be complemented with a physically‐based model (Pudasaini and Fischer, 2016) in the future. The extent to which a moraine dam is eroded strongly determines the availability of the solid fraction in the mixture downstream, and therefore the behaviour of the flow. The resulting variations in the solid content and the flow volume can exert manifold impacts on the mobility of the flow. Still, we do not expect threshold effects as strong as those inferred in Zone I.

Comparing the observed and the simulated impact area between the lakes Artizón Bajo and Jatuncocha, the maximum CSI value does not reach beyond 0.6, and the D2PC not below 0.15 (Table 4). A better performance is impeded by (i) an overestimate of lateral spreading and run‐up of the flow in the lower portion of Zone II; and (ii) an underestimate of the flow width over much of Zone III (Figures 10, 11, 12, 13).

Even though the result obtained in experiment E1 appears plausible (Figure 7) we have to keep in mind that it relies on an ad hoc parameter assumption, chosen towards confirmation of the observed patterns. This does not mean that the simulation would necessarily be fully valid. Instead, we can only confirm a certain degree of empirical adequacy in the sense of Oreskes *et al*. (1994), keeping in mind that: (i) other combinations of parameters may provide comparable results; and (ii) different simplifying or imperfect assumptions concerning the analytical model, the numerical approach and the parameterization may cancel out. Even though we can link the trends in the response of the simulation to the key inputs to our understanding of the related physical processes, the chosen parameters are unlikely to be physically correct.

Using r.avaflow allows back‐calculating complex hydro‐geomorphic process chains in an empirically adequate way. In contrast to most earlier approaches (Schneider *et al*., 2014; Schaub *et al*., 2016; Somos‐Valenzuela *et al*., 2016), r.avaflow supports a continuous simulation across the boundaries of the individual components of the process chain. Considering this a major step forward, we note that based on the present study there still remain important limitations for the predictive application of the model to possible future process chains for the following reasons:

1. Acknowledging that the uncertainties in the initial conditions (release mass, water content) and key flow parameters are to some extent aleatoric – particularly with regard to their spatial pattern – we depend on well‐founded empirical adequacy. The limitations of one‐at‐a‐time parameter studies, as applied in the present work, are well documented (Saltelli and Annoni, 2010). More appropriate approaches were proposed, e.g. by Formetta *et al*. (2015) or Fischer *et al*. (2015). Their application would be imperative, but represents a challenge due to the multi‐dimensional parameter space, including the spatial pattern in some parameters.
2. Considering what was said in (1), systematic back‐calculations of multiple events may lead to a set of guiding parameter ranges. Independent test cases would be necessary to confirm the general findings and to make a successful forward calculation more probable. Such an effort would be beyond the scope of the present article.
3. Some of the key characteristics of process chains are rarely documented (e.g. velocities and travel times), so that empirical adequacy has to remain incomplete. Well‐designed laboratory experiments could be employed in order to move as close as possible to an assessment of the physical adequacy of the simulation approach, e.g. with regard to the landslide–lake interactions.
4. The weakest link for forward predictions consists in the potentially inadequate assessment of the conditions above potentially impacted lakes, as even small misestimates of these conditions may lead to completely inappropriate results (threshold effects; Figure 11). Consequently, available resources should be put particularly in investigations on the characteristics of potential landslide release masses, and the material friction parameters in the upper most flow region.

While (1) to (3) apply to all efforts of spatial landslide modelling and beyond, but are particularly important when considering process chains, (4) is specific for process chains such as multi‐lake outburst floods. Due to the highly sensitive behaviour with regard to threshold effects such as lake overtopping, the assumption of fixed initial conditions and parameter values likely result in inadequate predictions. Consequently, forward predictions will, even more than for more common landslide processes, rely on: (i) the consideration of multiple scenarios; or (ii) the application of ranges of initial conditions and flow parameters instead of fixed values. The second of these results would result in likelihood measures such as impact indicator indices (Mergili *et al*., 2017) increasing the probability of successful forward simulations, and enabling the implicit consideration of uncertainties in the results (Krenn *et al*., 2016).

Consequently, a concerted communication process with decision makers is necessary to appropriately deal with uncertain simulation results. Targeting various groups of stakeholders and the public in a well‐adapted way will be essential for effective management of the risks associated with lake outburst floods and other types of process chains in the Cordillera Blanca and elsewhere in the world (Carey, 2005; Carey *et al*., 2012).

## Conclusions

Glacier retreat and the accompanying geomorphologic changes in the Artizón valley, Cordillera Blanca, Peru, have formed an environment favouring the development of a lake outburst cascade i.e. a steep and unstable lateral moraine slope geomorphologically connected to a system of glacial lakes. In 2012, a landslide from the left lateral moraine impacted the upper lake and induced a process chain proceeding kilometres downstream, entraining, transporting, and subsequently accumulating a significant amount of material. We have reconstructed the geomorphologic changes induced by the event from field observations and remotely sensed data, and attempted to back‐calculate the process chain with the two‐phase mass flow model employed within the computational framework r.avaflow. The design of r.avaflow enables continuous simulations across process boundaries, representing a major step forward. The results partly reveal the high sensitivity of process chain modelling to key inputs, such as release volume, basal friction or the entrainment coefficient. Due to multiple feedbacks and dependencies, these effects may even be stronger than for ‘ordinary’ mass flow processes. This is particularly true for the upper part of the chain, where the threshold effect of lake overtopping decides on the occurrence and characteristics of the flow downstream. With adequately chosen parameters, the documented and reconstructed patterns of the process chain are generally well reproduced by the r.avaflow calculations in terms of empirical adequacy, demonstrating the potential of computer simulations also for complex mass flow phenomena. However, our results indicate that the processes involved, and the input parameters and parameter combinations governing the simulation are not yet fully understood in their spatial distribution. Improved databases and thorough back‐calculations and parameter studies with a larger number of well‐documented events are needed to enable the anticipation of future events and their possible consequences in terms of scenario analyses or probability measures.
